# Special-Effect and Conventional Pigments in Black Light Art: A Multi-Technique Approach to an In-Situ Investigation

**DOI:** 10.3390/ma15196671

**Published:** 2022-09-26

**Authors:** Margherita Longoni, Serena Francone, Maddalena Boscacci, Diego Sali, Isabella Cavaliere, Vittoria Guglielmi, Silvia Bruni

**Affiliations:** 1 Department of Chemistry, University of Milan, Via C. Golgi 19, 20133 Milan, Italy; 2 Conservator of Contemporary Art and Polymateric Heritage, Museo delle Civiltà, Piazza G. Marconi 14, 00144 Rome, Italy; 3 Bruker Italia S.R.L. Unipersonale, Viale Vincenzo Lancetti 43, 20158 Milano, Italy

**Keywords:** fluorescent pigments, phosphorescent pigments, synthetic organic pigments, contemporary painting, in-situ analysis, Raman spectroscopy, spectrofluorimetry, visible reflectance spectroscopy

## Abstract

Since their introduction in the early decades of the 20th century, fluorescent pigments have found progressively wider applications in several fields. Their chemical composition has been optimized to obtain the best physical properties, but is not usually disclosed by the manufacturers. Even the other class of luminescent pigments, namely the phosphorescent ones, is now produced industrially. The peculiar optical properties of these pigments have attracted more and more the attention of famous artists since the middle of the last century. The Italian Black Light Art movement exploits the possibility of conveying different aesthetical messages depending on the kind of radiation (UV or visible) with which the artwork is illuminated. In the present work, a non-invasive in-situ investigation based on Raman, fluorescence, and visible-reflectance spectroscopies was performed on a series of Black Light Art paintings exhibited in Milan (Italy) in 2017, succeeding in the identification of the materials used by the artists. In particular, the use of both fluorescent and phosphorescent pigments, alone or combined with conventional synthetic organic pigments, has been recognized.

## 1. Introduction

Fluorescent paints were developed in the 1930s by the American brothers Robert and Joseph Switzer. Initially, such paints required irradiation with UV light to show their unique optical properties and were prepared using white shellac colored with fluorescent dyes. In the 1940s, the Switzer brothers succeeded in obtaining “daylight” fluorescent pigments, the emission of which can also be excited with visible light. At the end of the 1950s, a new process was patented, which made it possible to obtain pigments by combining fluorescent dyes with a new class of polymers, in particular a thermoplastic resin based on melamine–formaldehyde-*p*-toluene sulfonamide. It was thus possible to obtain fluorescent pigments with an adequate particle size, which could be used in paints similarly to traditional organic and inorganic pigments [1]. Daylight fluorescent pigments differ due to their very bright colors from those fluorescent pigments whose emission can only be excited by UV radiation. In fact, when irradiated with visible light, they give both reflected and emitted radiation as a response, leading to an apparent reflectance higher than 100%. The number of organic dyes used to produce fluorescent pigments is quite limited. Examples include Basic Violet 11, Basic Violet 10 (rhodamine B), Basic Red 1 (rhodamine 6G), Acid Red 52, Solvent Yellow 44, Basic Yellow 40, Solvent Yellow 135, and Solvent Yellow 160:1. By combining different dyes, however, a wide range of shades can be achieved in commercial pigments and paints [2].

Another class of “special effect” pigments is represented by phosphorescent materials, which, unlike the fluorescent ones, continue to emit even after the excitation source has been removed, giving the so-called “afterglow”. The first non-radioactive phosphorescent pigments were introduced in the 1930s and were based on copper-doped zinc sulfide (ZnS:Cu). Such pigments have a green afterglow with a maximum of around 520 nm and are still used nowadays [2]. In 1992 a new technology was developed which exploited alkaline earth metal (Sr, Mg, Ca) aluminates, usually doped with europium, to obtain brighter and longer-lasting phosphorescent pigments. The colors of their glow range from green-yellow to purple-blue. Another recent technology has made it possible to obtain a very pure sky-blue glow color, not available from alkaline earth aluminates, using alkaline earth silicates [3] instead.

Since their introduction, fluorescent pigments have found progressively wider applications in several fields, from advertising to safety signals, toys, sportswear, and automotive, while phosphorescent pigments are used for signaling in the dark, inks, coatings, plastics, silk-screen printing, etc.

Furthermore, the luminescent pigments, both fluorescent and phosphorescent, have attracted a great deal of attention from artists over the years. For example, the hypothesis of the use of phosphors in a painting was put forward concerning a Chinese painting described in the 11th century that looked different by day and by night [4]. In more recent times, the American painter Herb Aach, famous for his deep interest in color theory and who started using fluorescent pigments in the mid-1960s, wrote that “these pigments are at the frontier of color usage and color research” and that they “do not reflect normal color experiences, at least not in so far as everyday living is concerned”. In the same article, he also defines the concept of “metameric” color combinations, i.e., mixtures of two or more colors that give similar shades under the light source in which they were mixed but “may appear as different shades under another light” [5].

Since the 1960s, many American artists have used Day-Glo colors, such as Frank Stella, Andy Warhol, Roy Lichtenstein, Keith Haring, and Peter Halley. “Daylight” fluorescent paints are intended to be used for the realization of works to be observed under visible (natural and/or artificial) light. Black-light art was born when fluorescent colors were used for artworks to be observed under UV light: the first example known is *Ambiente spaziale a luce nera* (or *Black-light spatial environment*, 1949), an installation by the Italian artist Lucio Fontana. Always in Italy, in the early 1970s, Mario Agrifoglio began his research on metamerism with his geometric-abstract paintings. Similar technical research on metamerism has been carried out since the 1990s by LeoNilde Carabba, who also employs phosphorescent paints to obtain an additional version of her artworks, appreciable in total darkness [6].

It should be emphasized that the composition of commercial luminescent pigments, especially fluorescent ones, is usually not disclosed by manufacturers and, for this reason, several analytical techniques have recently been applied for the identification of their components. In particular, the techniques used include: spectrofluorimetry, both coupled with thin-layer chromatography (TLC) [7] and three-dimensional (3D) [8]; Raman spectroscopy with near-infrared excitation at 785 or 1064 nm [9,10,11]; surface-enhanced Raman spectroscopy (SERS) also coupled with TLC [11]; high-performance liquid chromatography coupled with mass spectrometry (HPLC-MS) [12,13]. Raman spectroscopy and HPLC-MS have also been applied to micro-samples taken from works of art to identify the materials used by the artists [9,12,13].

On the other hand, the in-situ investigation of paintings where these peculiar colors have been employed is challenging for several reasons. Of the techniques mentioned above, SERS and HPLC-MS, although very specific for identification purposes, cannot be applied non-invasively. Normal Raman spectroscopy is suitable for in-situ analysis, and its specificity has been demonstrated to recognize conventional synthetic organic pigments [14]. Still, it may, in principle, suffer from problems due to the fluorescence of the particular pigments considered here or other paint components, such as aged binders. Also, spectrofluorimetry allows for a non-invasive investigation and has already been successfully applied by some of us to the identification of conventional pigments and is also an example of fluorescent pigments in contemporary paintings [15,16]. It should be noted that in the case of fluorescent colors, a further element that complicates the analytical approach is represented by the very low quantities of dye contained in the pigment itself [17]. Additionally, artists could have mixed special-effect pigments with conventional ones, much more concentrated in paints, to achieve the desired metameric effect [6]. Despite this difficulty, identifying the coloring materials used by the artists is relevant from the point of view of the conservation and, if necessary, retouching of the paintings, as their aesthetical message should be preserved under the different light sources.

For the above reasons, in the present work, of which some preliminary results were anticipated in reference [18], a multi-technique approach based on Raman spectroscopy with SSE™ (Sequentially Shifted Excitation) instrumentation to reduce the fluorescence background, visible-excited spectrofluorimetry, and visible reflectance spectroscopy were adopted to investigate non-invasively the coloring materials used in three paintings by LeoNilde Carabba, one of the main representatives of the Italian Black Light Art movement as mentioned above, and in one painting by Maria Cristiana Fioretti, another contemporary Italian artist involved in the study of light as an expressive medium. The paintings, all dating back to the last decade, were examined during the exhibition “Black Light Art: la luce che colora il buio” held in 2017 at Palazzo Lombardia in Milano (Italy).

## 2. Materials and Methods

### 2.1. The Paintings

The paintings analyzed are made by two different artists. “Sisma” (“*Earthquake*”, 2017) was realized by Maria Cristiana Fioretti on the occasion of the exhibition and is composed of three canvases painted using fluorescent acrylic and oil colors. The technique does not involve significant color overlaps; the painting coats are mostly flat and applied directly to the ground layer. LeoNilde Carabba is the artist who made the other three paintings: “La stella polare e l’albero della vita” (“*The North star and the tree of life*”, 2010), “La grammatica del fuoco—Canto I” (“*The grammar of fire—Canto I*”, 2016) e “La grammatica del fuoco—Canto II” (“*The grammar of fire—Canto II*”, 2016). As mentioned above, in her artworks, traditional colors are present together with luminescent colors to obtain different metameric effects.

### 2.2. The Reference Materials

The paints analyzed as reference samples in the present work include all the colors of the Lefranc & Bourgeois Flashe Fluo series. In particular, they were Light Yellow (Y173), Green Yellow (G590), Bright Orange (O232), Grenadine Red (R376), Fire Red (R371), Bengal Red (R435), and Light Blue (B029).

Some authors had already analyzed the composition of these paints from the point of view of coloring materials by the FT-Raman, SERS, and TLC-SERS techniques [11]. Table 1 lists the analyzed materials, together with their coloring components.

To acquire the emission and visible reflectance spectra of these paints, in addition to the Raman spectra already reported in reference [11], they were spread both on canvas and glass slides.

### 2.3. Raman Spectroscopy

Raman analyses were performed in situ using a Bruker BRAVO handheld spectrometer, based on the patented SSE™ technology, which allows the mitigation of the fluorescence background and, consequently, to finally overcome the limits connected to the traditional Raman spectroscopy for the analysis of organic materials [19]. It is worth noting that, even for mock-up layers on canvas of the commercial reference colors, it was quite difficult to obtain pigment signals distinguishable from the fluorescence background when using a standard portable (or handheld) Raman spectrometer with 785 nm excitation while a satisfying spectral pattern could be acquired using the SSE™ Raman spectrometer (Figure S1, Supplementary Materials). This, therefore, was the instrument of choice for the investigation by Raman spectroscopy of these paintings.

BRAVO spectrometer includes two laser diodes (852 and 785 nm) to cover a broad spectral range (DuoLaser™ patented technology). The former collects the spectra from 300 cm^−1^ to 2000 cm^−1^, and the latter from 2000 cm^−1^ to 3200 cm^−1^. An appropriate algorithm allows data extraction showing a whole Raman spectrum in the full spectral range achievable, with an average spectral resolution of about 11 cm^−1^. The nominal laser power is less than 100 mW for both diodes. The beam is focused on a small rectangular area rather than a circular one to decrease the power density, reducing the risk of damaging the investigated artwork. The acquisition time and the number of coadds are automatically set by the instrument, with the possibility to use also a complete manual mode of operation. For the spectra acquired in the present study, the acquisition time typically ranged from 400 ms to 3 s and the number of coadds from 1 to 15.

### 2.4. Spectrofluorimetry and Visible-Reflectance Spectroscopy

Fluorescence and visible reflectance analyses were performed by a portable microprobe. The microprobe, equipped with an Olympus 20× objective (Tokyo, Japan), is connected via optical fibers to a halogen source (maximum power 150 W) and a Lot Oriel MS125 spectrometer (400 lines/mm grid, Darmstadt, Germany) provided with an Andor CCD detector (1024 × 128 pixels, Belfast, UK) cooled using a Peltier device. The wavelength calibration was based on the emission spectrum of a neon lamp.

For fluorescence analyses, the microprobe was equipped with an interference filter centered at 435 nm to select the excitation wavelength and a dichroic filter with a transmission range of 458–680 nm to eliminate from the spectrum the component due to the exciting radiation. The choice of visible exciting radiation was suggested by the higher selectivity it provides towards colored fluorescent materials. It is worth noting that in the case of commercial reference paints, the emission spectra obtained with visible exciting radiation are superimposable to those acquired with UV excitation at 365 nm [20]. Fluorescence spectra were collected as a sum of 10 scans with an exposure time of 0.2 s, and the power of the light source was adjusted according to the intensity of the signal obtained from each sample.

For visible reflectance analyses, the interference filter was removed, and the dichroic one was replaced by a beamsplitter 30/70 for the 400–700 nm spectral range. The reflectance spectra were acquired as the sum of 30 scans with an exposure time of 0.5 s, and the lamp’s power was set at 70 W. The analyses were preceded by acquiring a background spectrum in the absence of the incident radiation and a reference spectrum on a metal target coated with barium sulphate.

Both the fluorescence and visible reflectance spectra acquired on the paintings were compared with those of the reference materials using the correlation algorithm provided by the SpectralID function available in the GRAMS/AI software version 9.2 (Thermo Fisher Scientic Inc., Waltham, MA, USA). The algorithm estimates the similarity between the spectrum of the unknown (*Unkn*) and the spectra of the reference materials (*Lib*) based on a Hit Quality Index (HQI) defined as follows:

$$\mathrm{HQI}=1-\frac{{\left(Lib_{m}\cdot Unkn_{m}\right)}^{2}}{\left(Lib_{m}\cdot Lib_{m}\right)\left(Unkn_{m}\cdot Unkn_{m}\right)}$$

where

$$Lib_{m}=Lib-\frac{\sum_{i=j}^{n}Lib_{i}}{n}\;\;\;\mathrm{and}\;\;\;Unkn_{m}=Unkn-\frac{\sum_{i=j}^{n}Unkn_{i}}{n}$$

The highest similarity corresponds to a 0 value of HQI.

## 3. Results and Discussion

The results of the spectroscopic characterization of differently colored areas of the four paintings will be discussed in detail below based on each color as observed in visible light and are summarized in Table 2. The same table also shows the appearance of each measurement area in visible light, UV light (Wood’s lamp), and, for the three paintings by LeoNilde Carabba that the artist had intended for this further visual experience, also in the dark after UV irradiation with a Wood’s lamp and subsequent removal of the UV source. The chemical structures of the organic coloring substances, dyes, or pigments, identified in the works examined are reported in Table S1 (Supplementary Materials).

### 3.1. Red Colors

**Figure 1:**
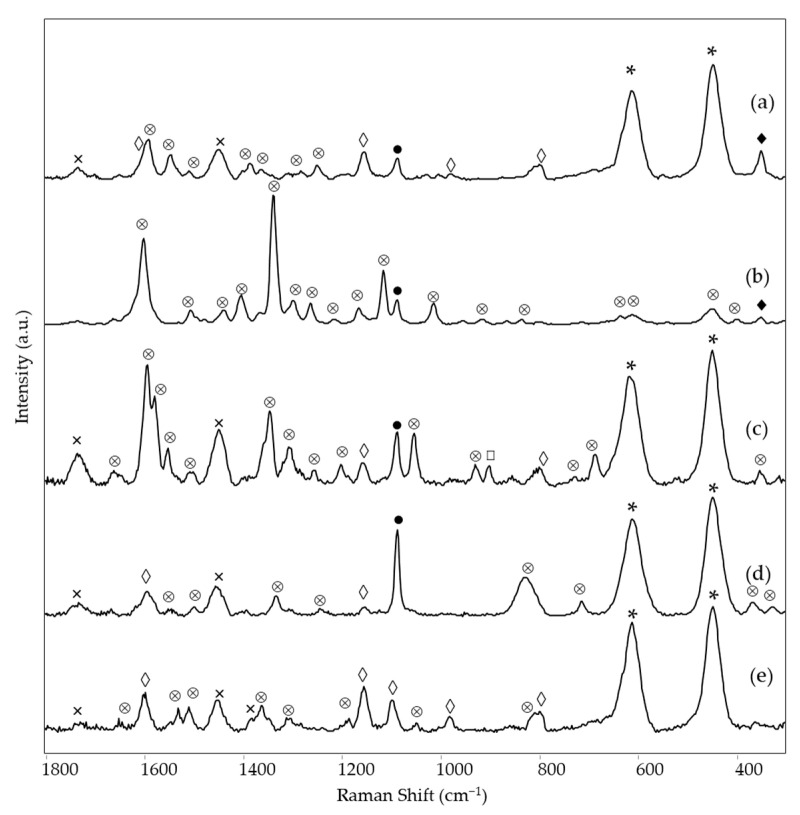
SSE™ Raman spectra of red areas: (a) *The grammar of fire—Canto I*, area 1, (b) *The grammar of fire—Canto II*, area 2, (c) *The North star and the tree of life*, area 1, (d) *The North star and the tree of life*, area 6 (orange) and (e) *Earthquake*, area 1. Legend: ⊗ = pigment (for wavenumbers and assignments see Table 2), * = rutile, ● = CaCO_3_, ◊ = resin, □ = Sr_2_MgSi_2_O_7_, ♦ = ZnS, × = binder (see text).

As shown in Figure 1, the red areas examined in the four paintings produced different Raman spectra for each.

In particular, in the case of *Earthquake*, the Raman spectrum of area 1 shows the typical bands of rhodamine 6G at 1647, 1530, 1510, 1360, 1344, 1309, and 1182 cm^−1^ [11] (Figure 1e and Table 2). The remaining Raman bands observed for the painting and also listed in Table 2 are due to: (i) the resin in which the rhodamine dye was dispersed to obtain the fluorescent pigment, corresponding to those reported in the literature [9] for a copolymer of *p*-toluene sulfonamide, melamine, and formaldehyde; (ii) the binder of the fluorescent paint, identifiable as polyvinyl acetate (PVA) thanks to the bands at 1728, 1450 and 1380 cm^−1^ [21]; (iii) titanium white in rutile form (intense bands at 608 and 444 cm^−1^, [22]; (iv) calcium carbonate as dolomite (band at 1098 cm^−1^) [23], probably present as an extender.

**Figure 2:**
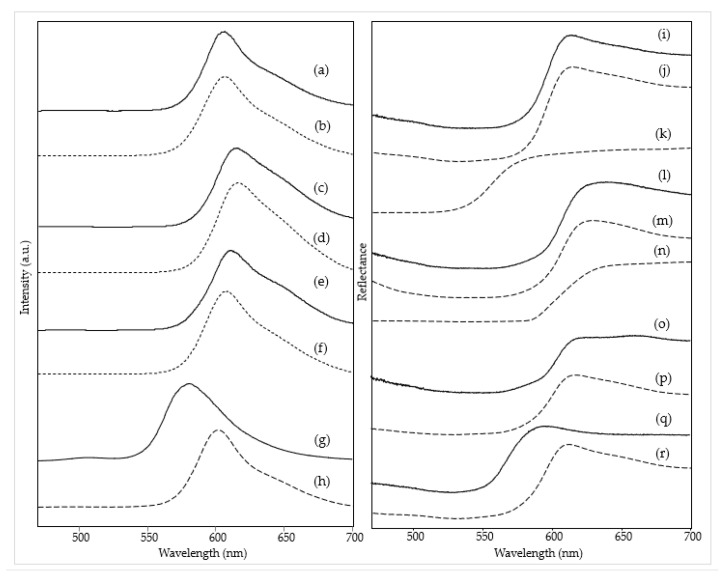
(**Left**) Emission spectra (λ_exc_ = 435 nm) of red areas: (a) *The grammar of fire—Canto II*, area 2, (b) reference sample of R371, (c) *The North star and the tree of life*, area 1, (d) reference sample of R435, (e) *Earthquake*, area 1, (f) reference sample of R376, (g) *The North star and the tree of life*, area 6 and (h) reference sample of O232. (**Right**) Visible reflectance spectra of red areas: (i) *The grammar of fire—Canto II*, area 2, (j) reference sample of R371, (k) reference sample of PO62, (l) *The North star and the tree of life*, area 1, (m) reference sample of R435, (n) reference sample of PR254, (o) *Earthquake*, area 1, (p) reference sample of R376, (q) *The North star and the tree of life*, area 6 and (r) reference sample of O232. Legend: (solid lines) spectra acquired on the paintings; (dashed lines) spectra of reference fluorescent paints or conventional pigments.

Consistently with the fluorescent pigment identified, the emission spectrum acquired on the same red area shows an excellent correspondence in the wavelength of the maximum (about 610 nm) and in the shape of the band with those among the reference paints considered here, which mainly contain rhodamine 6G and rhodamine B as dyes besides a small amount of yellow dye (Table 1), in particular Grenadine Red R376 (Figure 2e,f, left). In accordance with the proposed assignment, as reported in Table S2, the HQI obtained for this reference dye is very close to zero (0.028), while the HQI of the next hit of the library search, R371, is 0.044. On the other hand, considering the visible reflectance spectra of the red color in the painting and of the reference fluorescent red paint, the minimum (corresponding to the absorption maximum) and the inflection point of the curve are located about 6 nm higher for the painting than for the reference material (Figure 2o,p). The overall reflectance value is lower, probably due to the contribution of the underlying blue color (or a small amount of added blue color), presumably a phthalocyanine blue. Consistently, a rather high HQI value (0.318) was obtained for the spectrum of R376 and a comparable, even if slightly lower, one for R435 (Table S3). This pigment could also be responsible for the two reflectance minima around 630 and 700 nm [24] and a part of the band observed at 1530 cm^−1^ in the Raman spectrum [14].

In the case of *The grammar of fire—Canto I,* a more complex composition of the red color (area 1) could be recognized based on spectroscopic measurements. The Raman spectrum (Figure 1a) allows identifying the perinone orange pigment PO43 thanks to its bands at 1591, 1545, 1383, 1247, and 547 cm^−1^ (Table 2) [14]. A further Raman band is located at 348 cm^−1^ and is attributed to ZnS [25], most likely present as a phosphorescent pigment and therefore doped with Cu [2], although the Raman spectrum cannot provide positive evidence about the dopant. Part of the remaining bands can be assigned to the binder, which is most likely vinyl. Although, in this case, the diagnostic band of poly(vinyl) acetate around 1380 cm^−1^ is hidden by one of the strongest pigment signals, the rather strong band at ca. 840 cm^−1^ characterizing acrylic resins [26,27] is not observed. The typical signals of CaCO_3_ and rutile are recognizable, while bands are still present, possibly attributable to the resin component of a fluorescent paint at 1597, 1153, 978, and 798 cm^−1^ (Table 2). The fluorescent pigments are most likely of the rhodamine type, with an emission maximum of 612 nm (Figure S2, Supplementary Materials), while the visible reflectance spectrum was not acquired for this measurement area. A further emission spectrum was recorded after removing the UV source (Figure S2, Supplementary Materials) to eventually detect the phosphorescence signal and, despite a low signal-to-noise ratio, a maximum was observed around 620 nm, which is in agreement with the red glow showed by this area of the painting in the dark after UV irradiation. As zinc sulfide, i.e., the phosphorescent pigment identified through the Raman spectrum, has a green-yellow afterglow [2], the observed red emission is to be attributed again to the rhodamine-based fluorescent color, whose emission is excited, in the absence of an external source, by the radiation emitted by the phosphorescent pigment itself.

A similar combination of a conventional pigment, a fluorescent color, and a phosphorescent material was found in the red area 2 of *The grammar of fire—Canto II*. The Raman spectrum (Figure 1b) is almost exclusively dominated by the bands due to the benzimidazolone orange pigment PO62 [14] (Table 2), but the signal at 348 cm^−1^ associated with the presence of the phosphorescent pigment based on ZnS can also be observed. If the Raman spectrum does not provide any evidence about the fluorescent color, both the emission spectrum, with a maximum at 606 nm, and the visible reflectance spectrum, with a minimum of around 540 nm, show an almost perfect correspondence (*HQI* 0.009 and 0.031 respectively, Tables S2 and S3) with those of the reference color Fire Red R371 (Figure 2a,b left and 2i,j right), suggesting a rhodamine-based color again with a yellow component. In particular, the visible reflectance spectrum has the typical trend expected for a daylight fluorescent paint, with a maximum reflectance above 100% at a wavelength very close to the emission maximum (Figure 2i) [2].

Also, in the third painting by LeoNilda Carabba, *The North star and the tree of life*, the red area 1 was realized by mixing a conventional, a fluorescent, and a phosphorescent color. The first one is diketo-pyrrole-pyrrole red pigment PR254, recognized from its Raman bands, the strongest of which is located at 1592, 1577, 1553, 1343, 1304, and 1052 cm^−1^ (Figure 1c and Table 2) [14]. As in the cases discussed above, other Raman bands are linked to the binder, always assumed to be vinyl based on considerations similar to those reported for the red area of *The grammar of fire—Canto I*, calcium carbonate, and rutile. Similarly to the other two works by the same artist, the presence of a phosphorescent pigment can also be assumed from the Raman spectrum. In the present case, however, the diagnostic band is at 899 cm^−1^ (Table 2) and, as will be seen below with better evidence, can be assigned to Sr_2_MgSi_2_O_7_ [28], which, doped with europium, forms a phosphorescent pigment with a sky-blue afterglow (see Introduction) [29]. The presence of this pigment explains the bluish luminescence observed for this area of the painting after irradiation with a Wood’s lamp and subsequent removal of the UV source (Table 2). On the contrary, also concerning this painting, the fluorescent dyes responsible for the red emission observed under UV irradiation cannot be individuated from the Raman spectrum, where only two weak bands are observed due to the resin in which they are dispersed (Table 2). On the other hand, the fluorescence spectrum corresponds well (HQI 0.024, Table S2) for the band shape and the wavelength of the emission maximum (614 nm) to one of the reference paints, Bengal Red R435 (Figure 2c,d, left), whose components are rhodamine 6G and rhodamine B, without any part of yellow dye (Table 1). The visible reflectance spectrum also resembles that of the fluorescent reference color, with an HQI of 0.042 (Figure 2l,m, Table S3), but the maximum reflectance is less than 100%, and the trend in the red region of the spectrum is flatter, most likely due to the presence of the non-fluorescent pigment PR254 (Figure 2n).

Finally, also the orange hue of area 6 in the painting *The North star and the tree of life* will be considered in this section. This area does not belong to a homogeneous color layer but rather to darker touches on a yellow background, and this is reflected in the composition detected by Raman spectroscopy. From their Raman bands (Figure 1d), it can be assumed the presence of two pigments, namely the yellow inorganic bismuth vanadate BiVO_4_ (PY184, bands at 826, 363, and 321 cm^−1^ [30]) and much probably the aforementioned benzimidazolone orange PO62 (bands at 1594 and 1331 cm^−1^). As reported below, the same components are detected, albeit in a different proportion, in the yellow background painting. The remaining Raman bands are due, as usual, to white fillers and binder. Unlike the cases of the red areas already discussed, the emission and the visible reflectance spectra do not resemble any of the reference paints, in particular, the unique Bright Orange O232. The emission maximum is found at a significantly lower wavelength (580 versus 603 nm) than that of the reference paint (Figure 2g,h), with a quite high HQI of 0.510 (Table S2). A similar situation occurs for the visible reflectance spectrum, whose minimum and inflection point are significantly shifted at lower wavelengths (530 vs. 534 nm and 570 vs. 593 nm, respectively), with an HQI of 0.225, even if it always presents the characteristic trend expected for a daylight fluorescent pigment, with a reflectance value greater than 100% in the proximity of the emission maximum (Figure 2q,r). As a result, in this case it was impossible to make a hypothesis about the composition of the fluorescent paint.

### 3.2. Yellow Colors

**Figure 3:**
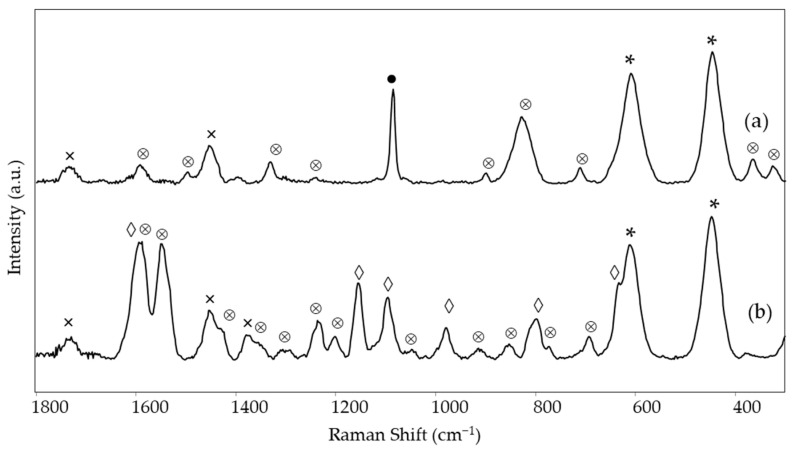
SSE™ Raman spectra of yellow areas: (a) *The North pole and the tree of life*, area 5 and (b) *Earthquake*, area 2. Legend: ⊗ = pigment (for wavenumbers and assignments see Table 2), * = rutile, ● = CaCO_3_, ◊ = resin, × = binder (see text).

The Raman spectrum obtained on the yellow area 2 of the painting *Earthquake* by Maria Cristiana Fioretti (Figure 3b) shows several bands in common with the spectrum of the reference fluorescent paint Light Yellow Y173 [11], among which those at 1588, 1548, 1429, 1235, 1203, and 692 cm^−1^ are characteristic of the coumarin dye Solvent Yellow 160:1 (Table 2), while the others are due to the melamine–formaldehyde-*p*-toluene sulfonamide resin. The bands due to the vinyl binder to calcium carbonate and rutile are also observed (Table 2).

**Figure 4:**
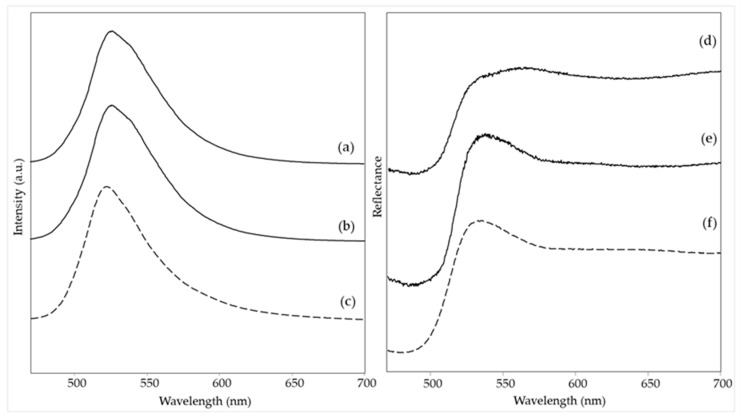
(**Left**) Emission spectra (λ_exc_ = 435 nm) of yellow areas: (a) *The North pole and the tree of life*, area 5, (b) *Earthquake*, area 2 and (c) reference sample of Y173. (**Right**) Visible reflectance spectra of yellow areas: (d) *The North pole and the tree of life*, area 5, (e) *Earthquake*, area 2, and (f) reference sample of Y173. Legend: (solid lines) spectra acquired on the paintings; (dashed lines) spectra of the reference fluorescent paint.

Despite the excellent correspondence between the Raman spectra, the emission maximum recorded for this measurement area is located at 526 nm, a wavelength approximately 7 nm higher than the reference yellow paint (Figure 4b,c), probably due to a greater concentration of pigment in the painting. as reflected in the corresponding HQI value of 0.193 (Table S2). The minimum reflectance in the visible region is observed at 488 nm, i.e., 5 nm above the reference paint, and the spectrum shows a very similar pattern (Figure 4e,f, Table S3).

As already highlighted for the red colors, even for the yellow ones, the paintings by LeoNilde Carabba are characterized by mixtures of pigments with different properties. In particular, a Raman spectrum very similar to the spectrum already discussed for the orange area 6 (see above) was obtained on the measurement area 5 of *The North star and the tree of life* (Figure 3a). Regarding conventional pigments, the bands attributable to BiVO_4_ (PY184) are observed, together with those possibly due to PO62 (Table 2), with a greater proportion of the former than the orange area. For the fluorescent component, a pigment similar to that of the yellow area of *Earthquake* can be assumed (HQI of Y173 equal to 0.098, Table S2), considering the correspondence of the two emission maxima at 526 nm (Figure 4a,b). Instead, as expected, due to the presence of the non-fluorescent pigment, the reflectance curve, whose minimum lies at 488 nm, i.e., the same wavelength observed for the yellow color of *Earthquake*, does not show the shape obtained when it is present only the fluorescent pigment (Figure 4d). Anyway, the HQI for the reflectance spectrum of Y173 is 0.114 (Table S3). Interestingly, the analyses did not suggest any phosphorescent component for the yellow area of *The North star and the tree of life*, which appears dark when the UV source is removed (Table 2).

### 3.3. Green Colors

The reference fluorescent green paint contains a fluorescent yellow pigment, based on SY160, and a non-fluorescent green pigment, PG7, which provides the desired shade (Table 1). The bands of the latter pigment dominate the Raman spectrum of the paint [11].

**Figure 5:**
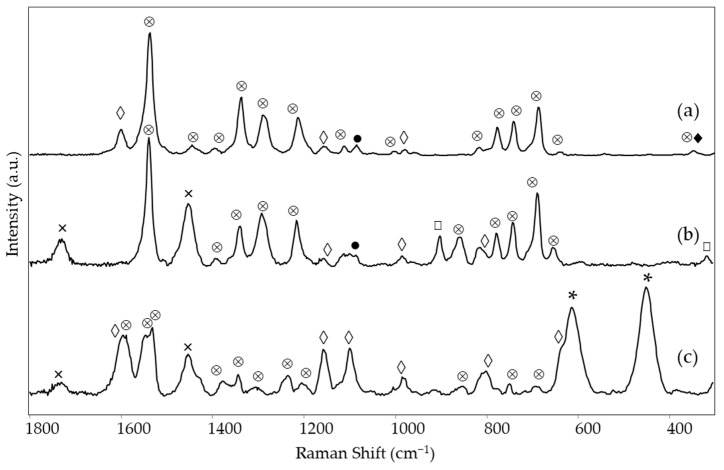
SSE™ Raman spectra of green areas: (a) *The grammar of fire—Canto I*, area 2, (b) *The North star and the tree of life*, area 4 and (c) *Earthquake*, area 3. Legend: ⊗ = pigment (for wavenumbers and assignments see Table 2), * = rutile, ● = CaCO_3_, ◊ = resin, □ = Sr_2_MgSi_2_O_7_, ♦ = ZnS, × = binder (see text).

In the green area 3 of *Earthquake*, on the contrary, the Raman spectrum demonstrates the presence of SY160, thanks to the bands coinciding with those already detected for the yellow area of the same painting (see above) (Figure 5c and Table 2). Furthermore, other Raman bands are observed at 1532, 1342, 746, and 680 cm^−1^ and can be assigned to phthalocyanine blue, possibly PB15:3 [14].

**Figure 6:**
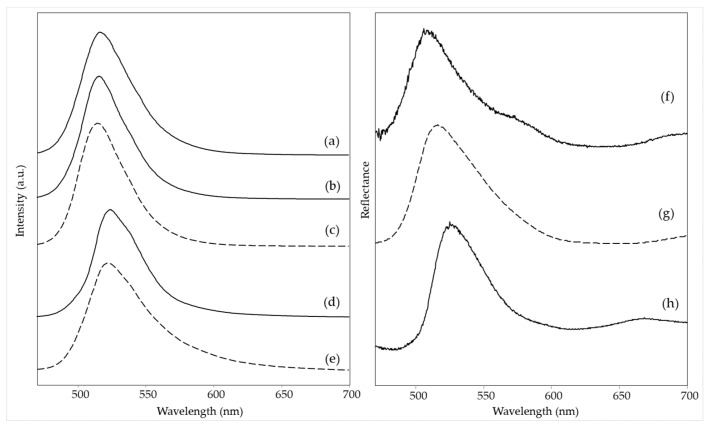
(**Left**) Emission spectra (λ_exc_ = 435 nm) of green areas: (a) *The grammar of fire—Canto I*, area 2, (b) *The North star and the tree of life*, area 4, (c) reference sample of G590, (d) *Earthquake,* area 3 and (e) reference sample of Y173. (**Right**) Visible reflectance spectra of green areas: (f) *The North star and the tree of life*, area 4, (g) reference sample of G590, and (h) *Earthquake*, area 3. Legend: (solid lines) spectra acquired on the paintings; (dashed lines) spectra of reference fluorescent paints.

Consistent with the Raman results, the fluorescence spectrum of the same green area of *Earthquake* has an emission maximum at 525 nm (Figure 6d), thus coinciding with those observed for the above discussed yellow colors containing presumably Solvent Yellow 160:1 and close to those of both the reference paints Y173 and, to a lesser extent, G590 (HQI 0.088 and 0.202 respectively, Table S2) (Figure 6c,e). At the same time, in the visible spectrum (Figure 6h), the reflectance minima, i.e., the absorption maxima, can be individuated around 620 and 706 nm, in accordance with the presence of PB15 [24] and 488 nm, due to the presence of the yellow fluorescent pigment. Consistently, the HQI for the reflectance spectrum of G590, which contains PG7 instead, is rather high, with a value of 0.381.

The green areas of two of the paintings by LeoNilde Carabba, i.e., area 2 of *The grammar of fire—Canto I* and area 4 of *The North star and the tree of life,* show very similar Raman spectra (Figure 5a,b and Table 2), where most bands, in particular those at 1535, 1337, 1287, 1210, 740 and 685 cm^−1^, can be assigned to the phthalocyanine green PG7 [31]. The main differences between the two spectra are related to the bands of the binder, very evident only for the measurement area of *The North star and the tree of life*, even if it is not possible to distinguish whether it is vinyl or acrylic, and to the bands of the phosphorescent components. In fact, for *The grammar of fire—Canto I*, a weak band is observed at 346 cm^−1^, suggesting the presence of a ZnS phosphor pigment, even if a contribution of PG7 to the same signal must also be taken into account. The hypothesis agrees with the green emission that persists for the green details of the painting after the removal of the UV source (Table 2), as ZnS:Cu pigments have an afterglow with a peak at 520 nm [2]. Instead, for *The North star and the tree of life*, the Raman bands at 899 and 314 cm^−1^ attributable to Sr_2_MgSi_2_O_7_ are well evident, indicating the presence of the blue phosphor already discussed above. This result is also in agreement with the bluish emission observed in the dark after UV irradiation, at least for some details of this area of the painting (Table 2).

The emission maxima at 515 nm for the same two areas are in accordance with the spectrum of the reference fluorescent green paint G590 (Figure 6a–c), with HQI values of 0.019 for area 2 of *The grammar of fire—Canto I* and 0.010 for area 4 of *The North star and the tree of life* (Table S2), thus attributing to Solvent Yellow 160:1 the green emission observed for those areas under UV irradiation. The same correspondence with the commercial paint is also observed for the visible reflectance spectrum (HQI 0.154, Table S3), which was acquired only for the green area of *The North star and the tree of life*. It shows minima at 640 and 718 nm, as expected due to the presence of PG7 [24], besides the minimum at 472 nm associated with the yellow component. In this case, it cannot be easily established if the phthalocyanine pigment is present in the paintings as a component of the fluorescent paint or used separately as a conventional color.

### 3.4. Blue Colors

The reference fluorescent paint for this color, Light Blue B029, is composed of phthalocyanine blue PB15 and a coumarin-based optical brightener. Only the first pigment contributes to the Raman spectrum [11], while the second is responsible for the emission at 460 nm observed with UV excitation (unpublished data).

**Figure 7:**
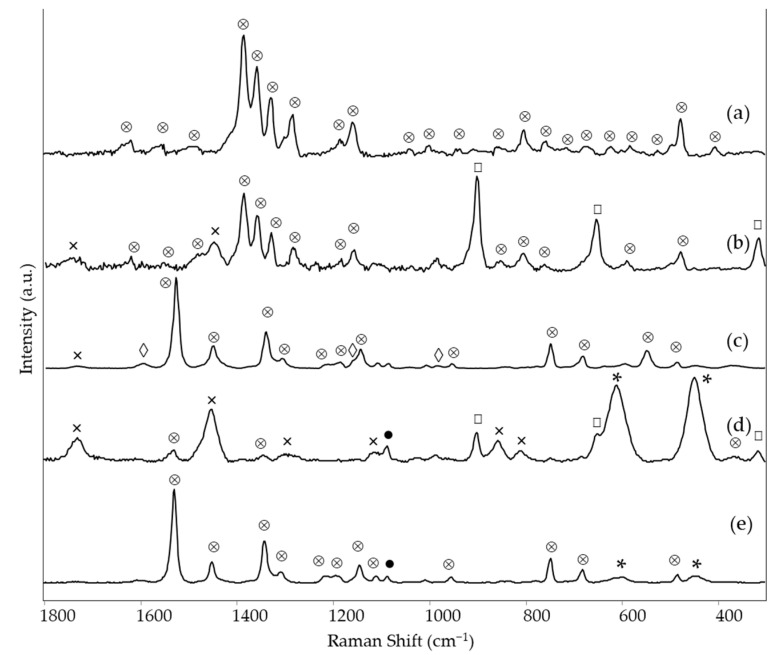
SSE™ Raman spectra of blue areas: (a) *The grammar of fire—Canto I*, area 3, (b) *The grammar of fire—Canto I*, area 4 (light blue), (c) *The grammar of fire—Canto II*, area 1, (d) *The North star and the tree of life*, area 2 (light blue) and (e) *The North star and the tree of life*, area 3. Legend: ⊗ = pigment (for wavenumbers and assignments see Table 2), * = rutile, ● = CaCO_3_, ◊ = resin, □ = Sr_2_MgSi_2_O_7_, × = binder (see text).

Of the blue areas examined, phthalocyanine blue PB15 could be identified based on the Raman spectra (Figure 7c,e and Table 2) in area 1 of *The grammar of fire—Canto II* and in area 3 of *The North star and the tree of life*. The main characteristic bands lie at 1527, 1450, 1341, 1144, 746, and 679 cm^−1^ [14]. For the first painting, however, also a band at 547 cm^−1^ is observed and assigned to ultramarine blue [22]. It was impossible to hypothesize about the binder, as only a weak band around 1734 cm^−1^ could be observed.

**Figure 8:**
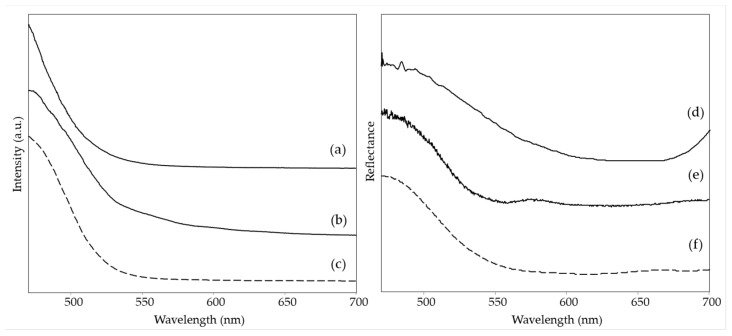
(**Left**) Emission spectra (λ_exc_ = 435 nm) of blue areas: (a) *The grammar of fire—Canto II*, area 1, (b) *The North star and the tree of life*, area 3, (c) reference sample of B029. (**Right**) Visible reflectance spectra of blue areas: (d) *The grammar of fire—Canto II*, area 1, (e) *The North star and the tree of life*, area 3, (f) reference sample of B029. Legend: (solid lines) spectra acquired on the paintings; (dashed lines) spectra of reference fluorescent paints.

For the same two areas, a fluorescence emission could also be detected (Figure 8a,b), even if, due to the instrumental configuration used, which contained a dichroic filter with a cut-on wavelength of 458 nm (see Section 2.4), it was not possible to determine exactly the wavelength of the emission maximum, which is equal or lower than 470 nm. Especially in the case of area 3 of *The North star and the tree of life*, however, the observed pattern of the emission curve has a very good correspondence with the reference fluorescent blue paint (Figure 8c), with an HQI of 0.061 (Table S2), suggesting that the material used by the artist has a very similar composition, both from the point of view of the conventional pigment, PB15, and the optical brightener. The reflectance spectrum (Figure 8e) shows minima at 630 and 715 nm, relative to phthalocyanine blue [24] and in accordance with the spectrum of the commercial blue paint (HQI 0.056, Table S3), and a further minimum at 560 nm, suggesting a red component. This can be explained considering that the examined area is quite inhomogeneous, and some touches of pink color (not analyzed in the present work) can be observed around the blue pattern (Table 2). The reflectance spectrum of area 1 of *The grammar of fire—Canto II* (Figure 8d) reflects the presence of ultramarine blue mixed with PB15, especially in the rise of the curve towards 700 nm [32]. As expected, a higher HQI value (0.235) for the reference paint B029 is obtained in this case (Table S3). Finally, consistent with the composition determined based on the spectroscopic data, neither of the two areas exhibits phosphorescence emission (Table 2).

For the blue area 3 of *The grammar of fire—Canto I*, a different pigment, namely indanthrone blue PB60, could be identified by its characteristic Raman bands at 1619, 1385, 1357, 1328, 1299, 1282, 1178, and 477 cm^−1^ [14] (Figure 7a and Table 2). No bands due to the binder were observed. This area showed no fluorescence emission when irradiated with UV light (Table 2).

Finally, two light blue areas, respectively in the painting just discussed *The grammar of fire—Canto I* (area 4) and in *The North star and the tree of life* (area 2), were considered, as they were both characterized by intense emission of blue phosphorescence after irradiation with UV light (Table 2). In both cases, the Raman bands at 899, 651, and 314 cm^−1^ (Figure 7b,d, Table 2) indicate the presence of the phosphorescent pigment based on Sr_2_MgSi_2_O_7_ [28]. The emission maximum observed in the visible spectrum acquired after UV irradiation and subsequent removal of the UV source is situated at 470 nm (Figure S2, Supplementary Materials), in accordance with the wavelength reported in the literature for the luminescence of a phosphor Sr_2_MgSi_2_O_7_ doped with Eu^2+^ [29]. The Raman spectrum of area 4 of *The grammar of fire—Canto I* also shows the typical bands due to PB60, most likely as the detail considered is composed of small dots on the blue background where the same pigment has been recognized (see above). For the light blue area of *The North star and the tree of life*, two weak bands at 1531 and 1343 cm^−1^ suggest the presence of a low amount of PB15, possibly also associated with fluorescent paint and thus explaining the bright blue color exhibited by this area in UV light, as in the case of the blue area 3 of the same painting. In this case, it is possible to assign to the binder the bands at 1730, 1451, and 1293 cm^−1^ and presumably also those at 1114, 858, and 812 cm^−1^, consistent with an acrylic resin [26]. Presumably, it is the binder of titanium white that, as demonstrated by the intensity of the corresponding Raman bands, is present in a considerable quantity in this light-colored area.

## 4. Conclusions

This work led to the identification of the coloring materials used in the four paintings investigated in a non-invasive manner during the exhibition dedicated to Black Light Art in Milano.

As regards the choice of materials by the artists, two different situations were encountered. On one hand, mainly fluorescent colors were used to obtain a painting with vivid tints in visible light and a different combination of colors under UV (“black”) light. This was the case of *Earthquake* by Maria Cristiana Fioretti, where the pigments used are based on a dye containing rhodamine (6G and B) for red and on a coumarin dye that coincides with or is very similar to Solvent Yellow 160:1 for yellow, while a mixture of the yellow fluorescent pigment and of phthalocyanine blue PB15 was recognized for green. Detecting the other typical components of the reference paints, namely the melamine–formaldehyde-*p*-toluene sulfonamide resin in which the dyes are dispersed and the poly(vinyl) acetate binder, reinforces the hypothesis that these were the colors most used by the artist.

On the other hand, a rather different and more complex situation was encountered for the coloring materials of the three paintings by LeoNilde Carabba. In fact, the works had to be exhibited in three different conditions, namely in visible light, in UV (“black”) light, and in the dark after removing the UV source. For this reason, fluorescent and phosphorescent paints were used by the artist. However, colors containing conventional pigments were also found not only in those details that should result in neither fluorescent nor phosphorescent, but also in areas where the special-effect pigments were present, probably to change the hue of the fluorescent paint and at the same time to reduce its psychedelic effect in visible light. Based on the emission spectra obtained on the paintings with visible excitation, the composition of the fluorescent colors can be assumed to be similar to the reference materials. In fact, for the red colors, rhodamine dyes have still been hypothesized. In the yellow and green areas, there must have been a dye similar to Solvent Yellow 160:1, and an optical brightener in those blue areas was found to be fluorescent. Concerning the phosphorescent materials, it has been possible to identify first the use of ZnS, most likely doped with Cu^2+^, to obtain a green afterglow, but also, at least in one case, to excite the emission in the dark of the fluorescent components. The other phosphorescent pigment that could be recognized was based on Sr_2_MgSi_2_O_7_ and characterized by a blue afterglow, which suggested it was doped with Eu^2+^. As conventional coloring materials, the synthetic organic pigment PB60 was identified in blue non-luminescent areas, while PR254, PO62, and PO43 mixed with fluorescent pigments were recognized in red areas and the inorganic pigment BiVO_4_ (PY184), also mixed with fluorescent paint, in yellow details. As previously discussed, the presence of the phthalocyanine pigments PB15 and PG7, respectively, in fluorescent blue and green areas could be due to the use of conventional colors or, to the same extent, of blue and green fluorescent paints that usually contain those pigments.

From a methodological point of view, the innovative non-invasive approach using the SSE™ handheld Raman spectrometer combined with visible-excited spectrofluorimetry proved effective for investigating these peculiar examples of contemporary painting and, more generally, of works where special-effect pigments have been used. Not requiring sampling, the proposed procedure can be useful for an extensive survey of this particular class of works of art, replacing or preceding traditional molecule-specific but invasive analyses and helping conservators and restorers define a preservation strategy.

## Figures and Tables

**Table 1:** Reference fluorescent paints and the coloring components recognized in their formulation [11].

Paint		Coloring Components
Light Yellow (Y173)	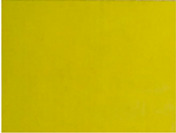	Solvent Yellow 160:1 [SY160]
Green Yellow (G590)	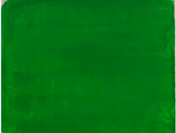	Phthalocyanine Green [PG7] Solvent Yellow 160:1 [SY160]
Bright Orange (O232)	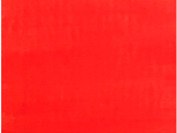	Rhodamine 6G [Rh6G] Rhodamine B [RhB] Solvent Yellow 160:1 [SY160]
Fire Red (R371)	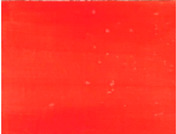	Rhodamine 6G [Rh6G] Rhodamine B [RhB] Solvent Yellow 160:1 [SY160]
Grenadine Red (R376)	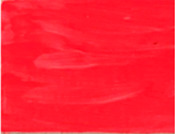	Rhodamine 6G [Rh6G] Rhodamine B [RhB] Solvent Yellow 160:1 [SY160]
Bengal Red (R435)	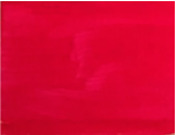	Rhodamine 6G [Rh6G] Rhodamine B [RhB]
Light Blue (B029)	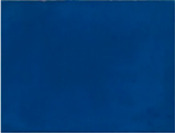	Phthalocyanine Blue [PB15] Coumarin-derivative optical brightener

**Table 2:** Measurement areas of the four paintings examined at the Black Light Art exhibition. For each area, the color, the wavenumbers of the Raman bands, and the wavelengths of the emission and visible absorption maxima are reported. For each color, the appearance in visible light, UV light (Wood’s lamp), and, when appropriate, in the dark after UV irradiation is shown. The materials identified based on the spectroscopic data are also indicated ^a^.

Painting				Raman Bands (cm^−1^) and Assignment ^b^		Emission Maximum (nm) and Assignment		Absorption Maximum (nm)
	Visible Light	UV Light	Dark after UV Irradiation					
*The Grammar of fire—Canto I*								
Red (1)	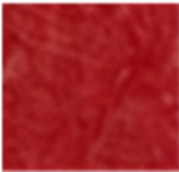	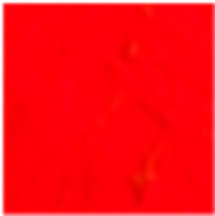	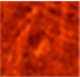	PO43	1591 m, 1545 w, 1383 w, 1247 w, 547 vw	Rhodamine-based fluorescent pigment (Rh6G + RhB) ^c^	612	n.a.
				ZnS (phosphorescent pigment)	348 m			
				CaCO_3_	1087 m			
				Rutile	610 vs, 444 vs			
				Resin	1597 sh, 1153 m, 978 w, 798 mw			
				Binder (PVA?)	2932 vs, 1732 w, 1445 m			
Green (2)	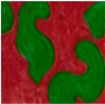	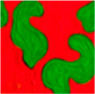	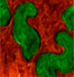	PG7	1535 vs, 1393 vw, 1337 m, 1287 m, 1210 m, 815 vw, 776 w, 740 m, 685 m, 346 vw	Fluorescent pigment based on SY160, or a similar dye	517	n.a.
				ZnS (phosphorescent pigment)	346 vw			
				CaCO_3_	1085 vw			
				Resin	1600 w, 1156 w, 1112 vw, 979 vw			
Blue (3)	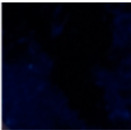	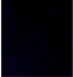	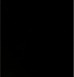	PB60	1619 m, 1558 w, 1493 w, 1385 vs, 1357 s, 1328 s, 1299 m, 1282 s, 1185 w, 1178 m, 1039 vw, 999 vw, 802 w, 757 vw, 678 sh, 622 vw, 582 vw, 524 vw, 496 vw, 477 m, 405 vw		-	n.a.
Light blue (4)	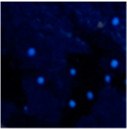	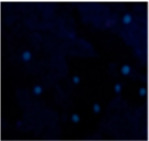	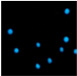	PB60	1620 vw, 1383 s, 1358 s, 1330 m, 1284 m, 1156 w, 803 w, 494 w, 477 w		n.a.	n.a.
				Sr_2_MgSi_2_O_7_ (phosphorescent pigment)	899 s, 651 m, 314 m			
				Binder (acrylic?)	2936 vs, 1737 w, 1450 w, 854 w)			
*The grammar of fire—Canto II*								
Blue (1)	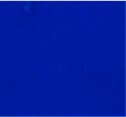	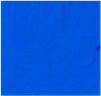	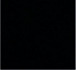	PB15	1527 vs, 1450 m, 1341 s, 1307 w, 1211 vw, 1186 vw, 1144 w, 1108 vw, 1006 vw, 850 vw, 828 vw, 775 vw, 746 m, 679 w, 597 vw, 482 w	Optical brightener	≤470	640
				Ultramarine blue?	547 m			
				Resin	1595 vw, 1157 sh, 982 vw			
				Binder (vinyl or acrylic?)	2940 m, 1734 vw			
Red (2)	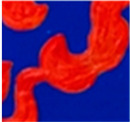	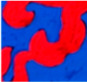	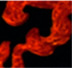	PO62	1662 vw, 1601 s, 1505 w, 1436 w, 1402 m, 1366 sh, 1336 vs, 1298 m, 1262 m, 1215 vw, 1164 w, 1115 m, 1011 mw, 952 vw, 913 vw, 863 vw, 835 vw, 635 vw	Rhodamine-based fluorescent pigment (Rh6G +RhB) ^c^	606	540
				CaCO_3_	1086 w			
				Rutile	608 w, 449 mw			
				ZnS (phosphorescent pigment)	347 w			
				Binder (vinyl or acrylic?)	2936 m, 1735 vw			
*The North star and the tree of life*								
Red (1)	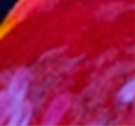	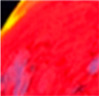	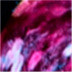	PR254	1664 w, 1592 vs, 1577 s, 1553 m, 1499 w, 1343 s, 1320 sh, 1304 m, 1254 w, 1201 w, 1052 m, 927 w, 725 vw, 684 w	Rhodamine-based fluorescent pigment (Rh6G +RhB)	615	550
				CaCO_3_	1086 m			
				Rutile	614 vs, 447 vs			
				Sr_2_MgSi_2_O_7_ (phosphorescent pigment)	899 w			
				Resin	1154 w, 795 w			
				Binder (PVA?)	1733 m, 1447 m			
Light blue (2)	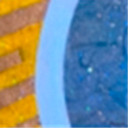	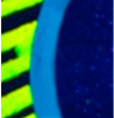	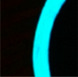	Sr_2_MgSi_2_O_7_ (phosphorescent pigment)	899 m, 651 sh, 314 w		n.a.	n.a.
				PB15	1530 w, 1343 w			
				CaCO_3_	1087 w			
				Rutile	609 s, 447 s			
				Acrylic binder	2937 vs, 2879 vs, 1730 m, 1451 s, 1293 w, 1114 w, 858 mw, 812 w			
Blue (3)	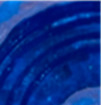	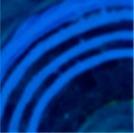	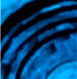	PB15	1527 vs, 1450 m, 1341 s, 1307 w, 1214 vw, 1192 vw, 1144 m, 1108 vw, 1006 vw, 850 vw, 828 vw, 775 vw, 746 m, 679 mw, 597 vw, 482 w	Optical brightener	≤470	560, 630, 715
				CaCO_3_	1085 w			
				Rutile	608 w, 447 w			
Green (4)	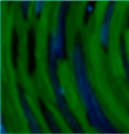	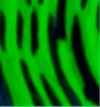	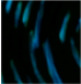	PG7	1537 vs, 1392 w, 1358 sh,1338 m, 1291 s, 1213 m, 775 m, 738 m, 684 s, 650 w	Fluorescent pigment based on SY160, or a similar dye	517	472, 640, 718
				Sr_2_MgSi_2_O_7_ (phosphorescent pigment)	899 m, 314 w			
				CaCO_3_	1084 w			
				Resin	1155 w, 892 w			
				Vinyl or acrylic binder	2932 vs, 1726 m, 1449 m			
Yellow (5)	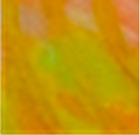	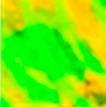	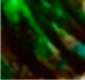	BiVO_4_ (PY184)	826 s, 363 w, 321 w	Fluorescent pigment based on SY160, or a similar dye	526	488
				Rutile	607 vs, 444 vs			
				PO62	1589 w, 1328 w			
				CaCO_3_	1085 vs			
				PVA	2932 vs, 1726 m, 1449 m, 1393 vw			
Orange (6)	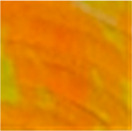	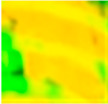	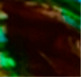	BiVO_4_ (PY184)	826 s, 363 w, 321 w	n.i.	580	530
				Rutile	608 vs, 444 vs			
				PO62?	1594 w, 1331 w			
				CaCO_3_	1085 vs			
				PVA	2932 vs, 1726 m, 1449 m, 1393 vw			
*Earthquake*								
Red (1)	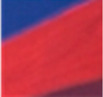	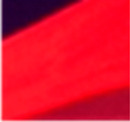		Rh6G	1647 vw, 1530 m, 1510 m, 1360 m, 1344 sh, 1309 mw, 1182 mw	Rhodamine-based fluorescent pigment (Rh6G + RhB)^c^	610	554
				Resin	1600 s, 1154 m, 1096 m, 977 w, 796 w			
				PVA	2931 vs, 1728 vw, 1450 m, 1380 w			
Yellow (2)	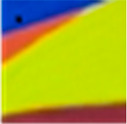	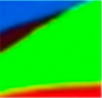		SY160	1588 s, 1548 s, 1429 sh, 1235 m, 1203 mw, 692 w	Fluorescent pigment based on SY160	526	488
				CaCO_3_	1086 s			
				Rutile	610 s, 447 vs			
				Resin	1593 s, 1154 m, 1094 m, 976 w, 798 w, 635 sh			
				PVA	2931 vs, 1733 vw, 1451 m, 1378 w			
Green (3)	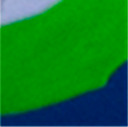	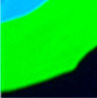		PB15	1532 s, 1342 m, 746 w, 683 w	Fluorescent pigment based on SY160	525	488, 620, 706
				SY160	1593 s, 1548 s, 1430 sh, 1236 w, 692 vw			
				Rutile	610 s, 447 vs			
				Resin	1593 s, 1154 m, 1094 m, 976 w, 798 w, 635 sh			
				PVA	2931 vs, 1733 vw, 1451 m, 1378 w			

^a^ n.a. = not acquired; n.i. = not identified. ^b^ vs = very strong: s = strong, sh = shoulder, m = medium, mw = medium weak, w = weak; vw = very weak. ^c^ with the addition of variable amounts of a yellow dye.

## Data Availability

Not available.

## Supplementary Materials

The following supporting information can be downloaded at: https://www.mdpi.com/article/10.3390/ma15196671/s1. Figure S1: Comparison between Raman spectra of fluorescent paints obtained with a portable spectrometer with 785-nm excitation and with the SSE™ handheld spectrometer; Figure S2: Emission spectra obtained with visible excitation of area 1 of *The grammar of fire—Canto I* and of the same area and area 2 of *The North star and the tree of life* in the dark after UV irradiation and removal of the UV source; Table S1: Chemical structures of the organic dyes or pigments identified in the paintings examined; Table S2: HQI of the first and second match obtained from the library search with the correlation algorithm on emission spectra; Table S3: HQI of the first and second match obtained from the library search with the correlation algorithm on reflectance spectra.
