# Supplementary material for: Special-Effect and Conventional Pigments in Black Light Art: A Multi-Technique Approach to an In-Situ Investigation
Source: Materials (Basel). 2022 Sep 26;15(19):6671. doi: 10.3390/ma15196671 (PMC9572826; doi:10.3390/ma15196671)
Supplement: Supplementary file 1 [file materials-15-06671-s001.zip › Figure captions SI.pdf]

Figure S1. (a) Raman spectra of a layer of the commercial Flashe Fluo color Bengal Red 435 on a glass slide, obtained respectively by a portable Raman microprobe with 785-nm excitation (grey line) and the SSE™ Bravo spectrometer (black line); (b) Raman spectra of a layer of the commercial Flashe Fluo color Light Yellow 173, obtained respectively by a portable Raman microprobe with 785-nm excitation (grey line) and the SSE™ Bravo spectrometer (black line).

Figure S2. (a) Emission spectrum (λ_{exc} = 435 nm) of *The grammar of fire - Canto I*, area 1; (b) emission spectrum of the same area after irradiation with a Wood's lamp and then removal of the UV radiation source; (c) emission spectrum of *The North star and the tree of life*, area 2, after irradiation with a Wood's lamp and then removal of the UV radiation source.
