# Supplementary material for: Special-Effect and Conventional Pigments in Black Light Art: A Multi-Technique Approach to an In-Situ Investigation
Source: Materials (Basel). 2022 Sep 26;15(19):6671. doi: 10.3390/ma15196671 (PMC9572826; doi:10.3390/ma15196671)
Supplement: Supplementary file 1 [file materials-15-06671-s001.zip › Table S1-S2-S3.pdf]

Table S1. Chemical structures of the organic dyes or pigments identified in the paintings examined.

Dye or pigment	Structure
Rhodamine 6G	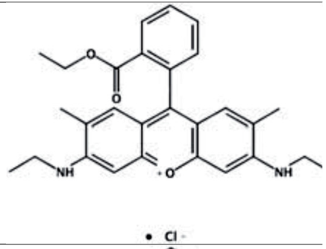
Rhodamine B	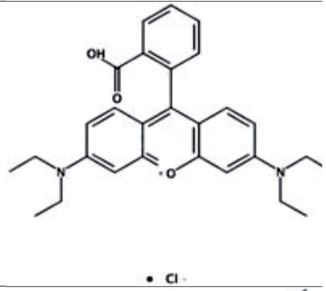
Solvent Yellow 160:1	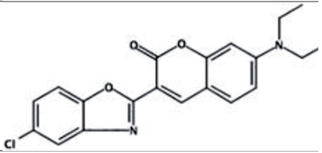
Pigment Orange 43	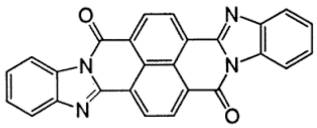
Pigment Orange 62	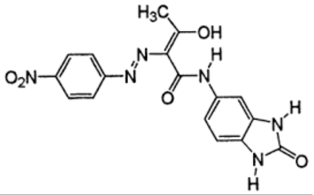
Pigment Red 254	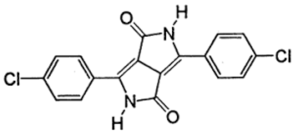
Pigment Green 7	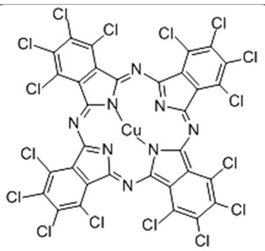

Pigment Blue 15	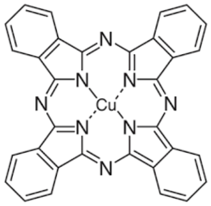
Pigment Blue 60	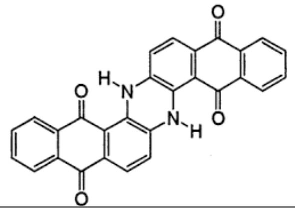

Table S2. HQI of the first and second match obtained from the library search with the correlation alghoritm on emission spectra.

	Area	HQI 1st	HQI 2nd
**Red and orange**	*The grammar of fire - Canto II*, red (2)	R371 0.009	R376 0.016
	The North star and the tree of life, red (1)	R435 0.024	R376 0.086
	Earthquake, red (1)	R376 0.028	R371 0.044
	The North star and the tree of life, orange (6)	O232 0.510	R371 0.620
**Yellow**	*The North star and the tree of life*, yellow (5)	Y173 0.098	-
	Earthquake, yellow (2)	Y173 0.193	-
**Green**	*The grammar of fire - Canto I*, green (2)	G590 0.019	Y173 0.038
	The North star and the tree of life, green (4)	G590 0.010	Y173 0.062
	Earthquake, green (3)	Y173 0.088	G590 0.202
**Blue**	*The grammar of fire - Canto II*, blue (1)	B029 0.091	-
	The North star and the tree of life, blue (3)	B029 0.061	-

Table S3. HQI of the first and second match obtained from the library search with the correlation alghoritm on reflectance spectra.

	Area	HQI 1st	HQI 2nd
**Red and orange**	*The grammar of fire - Canto II*, red (2)	R371 0.031	O232 0.034
	The North star and the tree of life, red (1)	R435 0.042	R376 0.102
	Earthquake, red (1)	R435 0.268	R376 0.318
	The North star and the tree of life, orange (6)	O232 0.225	R371 0.258
**Yellow**	*The North star and the tree of life*, yellow (5)	Y173 0.114	-
	Earthquake, yellow (2)	Y173 0.179	-
**Green**	*The North star and the tree of life*, green (4)	G590 0.154	-
	Earthquake, green (3)	G590 0.381	Y173 0.727
**Blue**	*The grammar of fire - Canto II*, blue (1)	B029 0.235	-
	The North star and the tree of life, blue (3)	B029 0.056	-
