# Supplementary figures and images for: Special-Effect and Conventional Pigments in Black Light Art: A Multi-Technique Approach to an In-Situ Investigation
Source: Materials (Basel). 2022 Sep 26;15(19):6671. doi: 10.3390/ma15196671 (PMC9572826; doi:10.3390/ma15196671)

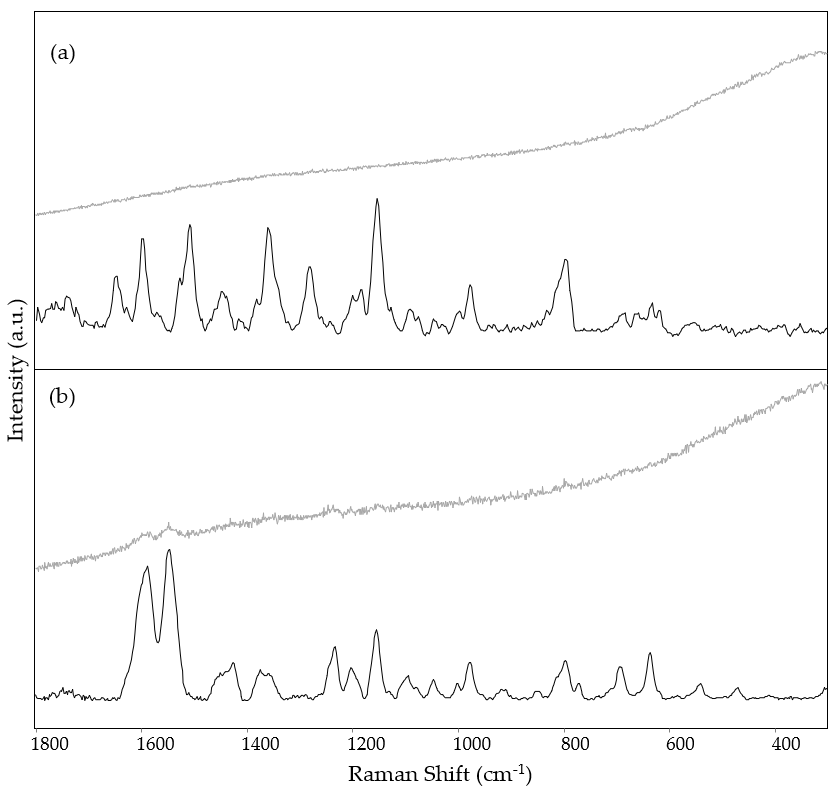

Supplement: Supplementary file 1 [file materials-15-06671-s001.zip › Figure S1.tif]

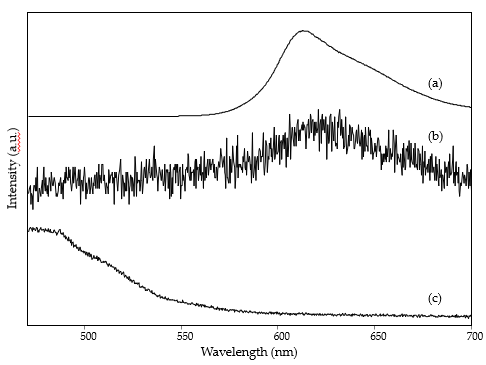

Supplement: Supplementary file 1 [file materials-15-06671-s001.zip › Figure S2.tif]
